# Di-(2-ethylhexyl) Phthalate Promotes Allergic Lung Inflammation by Modulating CD8α^+^ Dendritic Cell Differentiation *via* Metabolite MEHP-PPARγ Axis

**DOI:** 10.3389/fimmu.2022.581854

**Published:** 2022-05-19

**Authors:** Hsin-Han Tseng, Chia-Yang Li, Shin-Ting Wu, Hsiang-Han Su, Tzu-Hsuan Wong, Hsin-En Wu, Yu-Wei Chang, Shau-Ku Huang, Eing Mei Tsai, Jau-Ling Suen

**Affiliations:** ^1^Graduate Institute of Medicine, College of Medicine, Kaohsiung Medical University, Kaohsiung, Taiwan; ^2^Research Center for Environmental Medicine, Kaohsiung Medical University, Kaohsiung, Taiwan; ^3^Department of Laboratory, Taitung Hospital, Ministry of Health and Welfare, Taitung, Taiwan; ^4^National Institute of Environmental Health Sciences, National Health Research Institutes, Zhunan, Taiwan; ^5^Department of Medicine, Division of Allergy and Clinical Immunology, Johns Hopkins University School of Medicine, Baltimore, MD, United States; ^6^Department of Obstetrics and Gynecology, Kaohsiung Medical University Hospital, Kaohsiung, Taiwan; ^7^Department of Medical Research, Kaohsiung Medical University Hospital, Kaohsiung, Taiwan

**Keywords:** allergic lung inflammation, dendritic cell, PPAR γ, low-dose exposure, di-(2-ethylhexyl) phthalate, mono-(2-ethylhexyl) phthalate (MEHP)

## Abstract

Di-(2-ethylhexyl) phthalate (DEHP), a common plasticizer, is a ubiquitous environmental pollutant that can disrupt endocrine function. Epidemiological studies suggest that chronic exposure to DEHP in the environment is associated with the prevalence of childhood allergic diseases; however, the underlying causal relationship and immunological mechanism remain unclear. This study explored the immunomodulatory effect of DEHP on allergic lung inflammation, while particularly focusing on the impact of DEHP and its metabolite on dendritic cell differentiation and activity of peroxisome proliferator-activated receptor gamma (PPARγ). The results showed that exposure to DEHP at a human tolerable daily intake dose exacerbated allergic lung inflammation in mice. *Ex vivo* flow cytometric analysis revealed that DEHP-exposed mice displayed a significantly decreased number of CD8α^+^ dendritic cells (DCs) in spleens and DC progenitors in the bone marrow, as well as, less interleukin-12 production in splenic DCs and increased T helper 2 polarization. Pharmacological experiments showed that mono-(2-ethylhexyl) phthalate (MEHP), the main metabolite of DEHP, significantly hampered the differentiation of CD8α^+^ DCs from Fms-like tyrosine kinase 3 ligand-differentiated bone marrow culture, by modulating PPARγ activity. These results suggested that chronic exposure to DEHP at environmentally relevant levels, promotes allergic lung inflammation, at least in part, by altering DC differentiation through the MEHP-PPARγ axis. This study has crucial implications for the interaction(s) between environmental pollutants and innate immunity, with respect to the development of allergic asthma.

## Introduction

The prevalence of allergic diseases has dramatically increased worldwide, especially in industrialized countries, for unknown reasons (1). Allergic diseases constitute a substantial public health concern and an economic burden. One explanation for the high prevalence of allergic diseases in industrialized countries is the observed dysregulation of immune development upon long-term exposure to compounds that are classified as environmental endocrine disruptors (2, 3). Epidemiological studies have shown associations between exposure to phthalates, particularly di-(2-ethylhexyl) phthalate (DEHP), and the risk of developing allergies and asthma in children (4, 5). However, the mechanisms of action remain unclear, and no causal relationship has been established.

DEHP, a widespread environmental contaminant and endocrine disruptor, is used principally as a plasticizer for a wide range of purposes, in both developed and developing countries (6). The major route of DEHP exposure is from food, owing to the wide use of this compound in the manufacture of food packaging and containers (7). Notably, DEHP has been declared illegal for use as a food additive in Taiwan (8). In humans, exposure to DEHP results in detectable concentrations of several of its metabolites in the urine, including the hydrolytic metabolite mono-(2-ethylhexyl) phthalate (MEHP) and the two oxidized metabolites mono-(2-ethyl-5-hydroxyhexyl) phthalate and mono-(2-ethyl-5-oxohexyl) phthalate (9, 10). At present, the level of DEHP exposure in the general population appears to be lower than the tolerable daily intake (TDI; 50 μg/kg body weight [BW] per day), based on calculations made for the aforementioned urinary metabolites (11, 12). However, long-term exposure to DEHP still poses a health concern, because it has anti-androgenic activity through an unidentified receptor and has been associated with reproductive abnormalities, neurological defects, and the development of certain tumor types (13, 14). In addition, DEHP altered the function of human plasmacytoid dendritic cells (pDCs) *in vitro* (15). Iatrogenic DEHP exposure affected gut microbiota pattern and vaccine response in newborns (16). These studies suggest that DEHP may have an adverse impact on immune responses or development in humans.

Dendritic cells (DCs) are the key innate cell type that link innate and adaptive immunity, and DC dysregulation promotes the development of allergic diseases, as DCs instruct the differentiation of allergen-specific T helper 2 (Th2) cells, and in turn, lead to lung inflammation and remodeling (17, 18). CD8α^+^ cDC is the major interleukin (IL)-12-producing cDC subset, and blocking the differentiation of this subset skews immunity to the Th2 response (19, 20). Our recent study demonstrated that transmaternal DEHP exposure aggravates allergic lung inflammation in murine offspring, by prominently increasing apoptosis in CD8α^+^ cDCs (21). It has been demonstrated that MEHP, the primary metabolite of DEHP, can activate the activity of peroxisome proliferator-activated receptor gamma (PPARγ) in cell transactivation assays (22), and that PPARγ mediates the cross-talk with other nuclear receptors (23–25), thus affecting DC function (26, 27) and differentiation (28), as well as, T-cell lipid metabolism (29, 30). Furthermore, PPARγ upregulation enhances the migration and Th2-priming capacity of lung DCs, suggesting a pro-inflammatory role for PPARγ in Th2-mediated allergic lung inflammation (31). However, a previous study suggested an anti-inflammatory role for PPARγ, in that PPARγ-activated DCs contribute to the development of CD4^+^ T-cell anergy (32). Furthermore, PPARγ activation in macrophages has anti-inflammatory effects (33). In addition, systemic treatment with a pharmacological PPARγ agonist dampens inflammation, at least in part by inhibiting DC function in various inflammatory diseases, including asthma (34). Although it has been shown that environmental levels of DEHP (30 µg/kg BW/day) exacerbate ovalbumin (OVA)-induced murine asthma (35), it remains largely unclear whether chronic exposure to low-dose DEHP can enhance allergic lung inflammation mainly through DCs. In addition, it also needs to be determined whether DEHP/MEHP-conditioned DCs play a pro-inflammatory role or inhibitory role, in a PPARγ-dependent manner.

To address these important issues, we explored the potential pathogenic role of DEHP in the development of allergic asthma and its underlying mechanism. We established an OVA-induced allergic lung inflammation model in mice, under conditions mimicking human exposure levels and routes for DEHP. The results demonstrated that chronic exposure to DEHP at environmentally relevant levels exacerbates allergic lung inflammation, by altering cDC differentiation in a PPARγ-dependent manner.

## Material and Methods

### Mice and Phthalate Exposure

Female BALB/c mice (6–8 weeks of age) were obtained from the National Laboratory Animal Center, Taiwan. All mice were maintained at the Animal Center of Kaohsiung Medical University, which is internationally accredited by the Association for Assessment and Accreditation of Laboratory Animal Care. The protocol used in all animal experiments was approved by the Institutional Animal Care and Use Committee of Kaohsiung Medical University (permit numbers: 104115, 109075) and was carried out in accordance with the guidelines and regulations of the institution.

**Figure 1:**
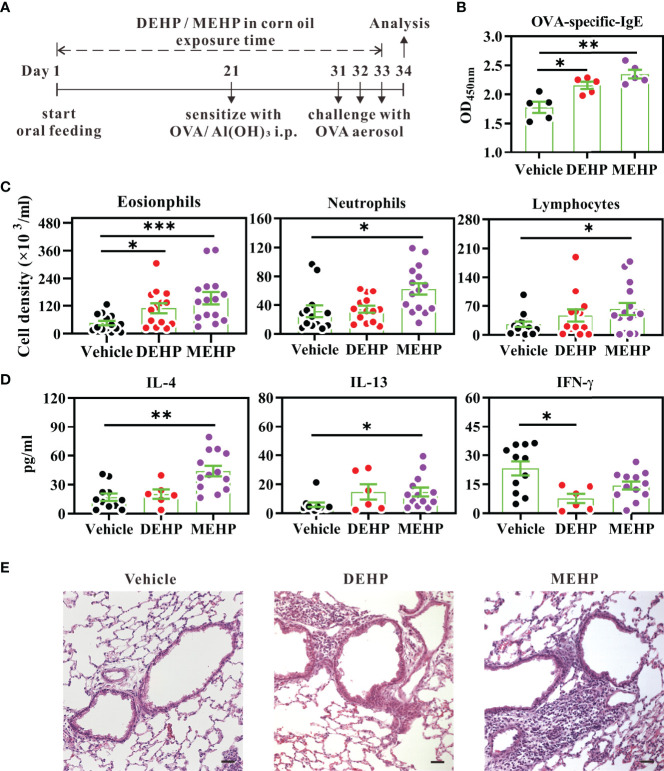
Effect of DEHP exposure on allergic lung inflammation in a BALB/c mouse model of mild asthma. **(A)** Schematic depiction of the model of OVA-induced asthma, upon exposure to DEHP or MEHP. BALB/c mice were given oral DEHP or MEHP daily, at a dosage of 37 μg/kg BW, and all mice were immunized and challenged with OVA. **(B)** Serum levels of OVA-specific IgE (10-fold dilution) were determined using ELISA. Cell subsets **(C)** and cytokine levels **(D)** in BALF were analyzed using flow cytometry and ELISA, respectively. **(E)** Representative lung sections stained with hematoxylin and eosin. Scale bar: 50 μm. Results have been represented as mean ± SEM. *P < 0.05, **P < 0.01, and ***P < 0.001 *vs.* vehicle (as assessed using Mann–Whitney U test). In **(B)** n = 5 mice per group; in **(C, D)** n = 14 or 15 mice per group in four independent experiments; in **(E)** data are representative of two independent experiments.

To mimic the exposure that humans typically encounter in daily life, female mice were administered a daily oral gavage of DEHP or MEHP (AccuStandard, New Haven, CT, USA) at the human TDI dose determined by the EU Scientific Committee for Toxicity, Ecotoxicity, and the Environment (36), which is 37 μg DEHP/kg BW, for a study period of 34 days. After the initial administration of phthalate for 20 consecutive days, the mice were immunized with OVA, to induce mild allergic lung inflammation (**Figure 1A**). Control mice were orally administered 0.33% ethanol in corn oil, as a vehicle control. For bone marrow cell *ex vivo* analysis, the mice received a daily oral gavage of DEHP, at a dosage of 37 μg DEHP/kg BW, or vehicle for 10 d.

### Model of Allergic Lung Inflammation

The mice were sensitized to OVA *via* peritoneal injection of 0.2 ml sterile saline containing 20 μg OVA (Sigma-Aldrich, St. Louis, MO, USA) and 50 μl aluminum hydroxide hydrate adjuvant (Thermo Fisher Scientific, Waltham, MA, USA). Ten days after sensitization, the mice were challenged with 3% OVA aerosol for 20 min, for three consecutive days. On the day after the last challenge, cell subsets and cytokine levels in the bronchoalveolar lavage fluid (BALF) were analyzed using multi-parametric flow cytometry and enzyme-linked immunosorbent assay (ELISA), respectively. Blood samples were collected before sacrifice, and the sera were analyzed for the presence of anti-OVA IgE (**Figure 1A**).

### Flow Cytometry

For analysis of immune cells in BALF, the cells were stained with FITC-conjugated anti-MHC class II (M5/114.15.2; eBioscience, Northern Ireland, UK), PE-conjugated CCR3 (83101; R&D Systems, Minneapolis, MN, USA), Alexa Fluor™ 700–conjugated anti-CD3 (17A2; BD Biosciences, San Diego, CA, USA), and anti-B220 (RA3-6B2; eBioscience) monoclonal antibodies. Splenocytes were stained with FITC-conjugated anti-CD8α (53-6.7; eBioscience), PE-conjugated anti-PDCA-1 (129C1; BioLegend, San Diego, CA, USA), PerCP-cy5.5-conjugated anti-CD4 (RM45; BioLegend), APC-conjugated anti-CD11c (N418; BioLegend), Alexa Fluor™ 700-conjugated anti-MHC class II (M5/114.15.2), and LIVE/DEAD™ Fixable Red (Invitrogen, Carlsbad, CA, USA).

For bone marrow DC progenitor analysis, bone marrow cells were stained with eF450-conjugated anti-CD45 (30-F11; eBioscience), PE-conjugated anti-CD4 (RM4-5), PE-conjugated anti-CD8α (53-6.7), PE-conjugated anti-CD49 (DX5; eBioscience), PerCP-cy5.5-conjugated anti-CD19 (1D3/CD19; BioLegend), APC-conjugated anti-CD11c (N418), Alexa Fluor™ 700-conjugated anti-MHC class II (M5/114.15.2), and LIVE/DEAD™ Fixable Red. After washing, the cells were intracellularly stained with PPARγ rabbit mAb (C26H12; Cell Signaling Technology, Danvers, MA, USA), followed by FITC-conjugated goat anti-rabbit IgG (H+L) secondary antibody (F2765; Invitrogen), in the presence of the Transcription Factor Buffer Set (BD Biosciences). Flow cytometry (LSRII; BD Biosciences) was used to carry out the phenotype analysis of immune cells in the bone marrow or spleen, as well as, to analyze the cellular composition of BALF.

The absolute number was determined by multiplying the percentage of a given subset in viable cells by the total cell count of the samples.

### ELISA for OVA-Specific IgE

The levels of OVA-specific IgE in sera were determined using ELISA. Briefly, 10-fold diluted serum (100 μl for each sample) was added to the wells of an OVA-coated ELISA plate (NuncMaxiSorp™ flat-bottom, Thermo Fisher Scientific), followed by addition of biotinylated rat anti-mouse IgE (R35-72, 2 μg/ml, BD Biosciences), horseradish peroxidase-conjugated avidin (40-fold dilution, BD Biosciences), and NeA-Blue (tetramethylbenzidine substrate; Clinical Science Products, Mansfield, MA, USA). The reaction was stopped by the addition of 3 N H_3_PO_4_, followed by measurement of the absorbance using a VersaMax™ ELISA reader (Molecular Devices, Temecula, CA, USA) at the wavelength of 450 nm, corrected to the absorbance at the wavelength of 540 nm.

### Histology

Whole lungs were fixed in 10% neutral-buffered formalin for 24 h, dehydrated, and embedded in paraffin. Tissue sections (3 μm thickness) were stained with hematoxylin and eosin, according to the manufacturer’s protocol (Novolink™ Polymer Detection Systems, Leica, Newcastle, UK). Images of the stained tissues were captured using a TissueFAXS Imaging System (TissueGnostics, Vienna, Austria).

### CD4^+^ T-Cell Differentiation

For splenic cDC purification, splenocytes were labeled with CD11c-microbeads (Miltenyi Biotec, Sunnyvale, CA, USA) for positive selection, according to the manufacturer’s instructions (autoMACS^®^ Separator, Miltenyi Biotec, Bergisch Gladbach, Germany). Naïve CD4^+^ T cells were purified using a CD4^+^CD62L^+^ T Cell Isolation Kit (Miltenyi Biotec), according to the manufacturer’s instructions. Purified naïve CD4^+^ T cells were co-cultured with splenic cDCs for 5 d, in RPMI-1640 containing 10% FBS supplemented with anti-mouse CD3 (1 μg/ml) and recombinant mouse IL-2 (10 ng/ml, R&D Systems). On day 5, the cells were re-stimulated with a cell activation cocktail (BioLegend) and monesin (2 μM, BioLegend), for a further 4 h. The cells were then stained with PE-conjugated IL-4 (11B11, BD Biosciences) and APC-conjugated interferon gamma (IFN-γ; XMG1.2, BD Biosciences) using a Fixation/Permeabilization Kit (BD Biosciences), according to the manufacturer’s instructions.

### Fms-Like Tyrosine Kinase 3 Ligand (Flt3L)-Differentiated Dendritic Cell (FL-DC) Treatment

FL-DCs were prepared as previously described (37), with some modifications. In brief, bone marrow cells were cultured at a concentration of 3×10^5^ cells/ml in RPMI-1640 medium containing 10% FBS supplemented with 200 ng/ml recombinant murine Flt3L (rmFlt3L, PeproTech, Rocky Hill, NJ, USA) and 2-mercaptoethanol (50 μM, Sigma-Aldrich), for 8 d. Cells were treated with MEHP, GW9662 (PPARγ antagonist; Tocris, Bristol, UK), or GW1929 (PPARγ agonist; Sigma-Aldrich) at various concentrations or with 0.1% ethanol (vehicle control) at the beginning of day 1 of culture. The medium containing rmFlt3L and/or chemicals or 0.1% ethanol was refreshed on days 4 and 6. On day 8, the cells were stained with the following antibodies, for phenotype analysis using flow cytometry: PE-conjugated anti-CD86 (GL-1; eBioscience), PerCP-cy5.5-conjugated anti-CD24 (M1/69; BioLegend), BV421-conjugated anti-CD45RA (14.8; BD Biosciences), APC-conjugated anti-CD11c (N418), APCcy7-conjugated anti-CD11b (M1/70; BioLegend), Alexa Fluor™ 700-conjugated anti-MHC II (M5/114.15.2), and LIVE/DEAD™ Fixable Red. For functional analysis, day-8 FL-DCs were washed and then stimulated with CpG1826 (10 μg/ml, InvivoGen, Carlsbad, CA, USA) for 24 h. The supernatants of FL-DCs were assessed for levels of cytokines using ELISA (R&D Systems).

### Western Blot

Day-3 Flt3L-cultured bone marrow cells were treated with MEHP, GW1929, and/or GW9662 for 6, 18, or 24 h. Cells treated with 0.1% ethanol were used as the vehicle control. Western blot analysis was performed as described previously (38). Briefly, harvested cells were lysed using radioimmunoprecipitation assay buffer supplemented with protease inhibitors (Sigma-Aldrich). The BCA Protein Assay Kit was used to determine the concentration of the cell lysate samples, according to the manufacturer’s instructions (Thermo Scientific). Equal amounts of proteins were resolved using sodium dodecyl sulfate-polyacrylamide gel electrophoresis (SDS-PAGE) and detected by means of immunoblotting with antibodies against fatty acid-binding protein 4 (FABP4; TA328110, OriGene, Rockville, MD, USA), PPARγ (H-100, Santa Cruz Biotechnology, Dallas, TX, USA), and β-actin (A2228, Sigma-Aldrich). The signals were visualized using an ECL chemiluminescence substrate (Thermo Scientific), and the band intensities were quantified using the ChemiDoc XRS+ System (Bio-Rad, Hercules, CA, USA).

### Statistical Analysis

The non-parametric Mann–Whitney U test was used to compare continuous variables between two groups. One-way ANOVA followed by Dunnett’s multiple comparison test was used to compare differences among three or more groups. All statistical tests were performed using Prism 9.0 (GraphPad, San Diego, CA, USA). Statistical significance was set at P<0.05.

## Results

### Chronic, Low-Level Exposure to DEHP Enhances OVA-Induced Allergic Lung Inflammation in Mice

To simulate the exposure of humans to environmental DEHP, BALB/c mice were administered daily oral doses of DEHP or its major metabolite MEHP (37 μg/kg BW per day) during the entire study period. After the initial 20-day exposure, the mice were sensitized and challenged with OVA, to induce allergic lung inflammation (**Figure 1A**). The selected dose of DEHP was based on the previous human TDI determined by the EU Scientific Committee for Toxicity, Ecotoxicity, and the Environment (36), which is essentially midway between the estimated total daily oral intake of Denmark children aged 1–6 years (26 μg/kg BW per day) and the current human TDI (50 μg/kg BW per day), as recommended by the European Food Safety Authority (39). Thus, the dose of 37 μg DEHP/kg BW per day used here can be considered an environmentally relevant exposure level for humans.

As shown in **Figures 1B, C**, exposure to DEHP significantly enhanced the titer of OVA-specific IgE and eosinophil numbers in the BALF. In addition, the IFN-γ level in the BALF of DEHP-treated mice was significantly lower than that in the control mice (**Figure 1D**). Furthermore, upon exposure at the same oral dose, the principal DEHP metabolite MEHP had a more profound effect on allergic lung inflammation than DEHP, including increased infiltration of eosinophils, neutrophils, and lymphocytes, as well as, higher levels of IL-4 and IL-13 (**Figures 1C, D**). Histopathology revealed that exposure to DEHP and MEHP substantially increased the infiltration of inflammatory cells into the lungs, as compared to exposure to vehicle controls (**Figure 1E**). These data suggested that DEHP, which quickly degrades into MEHP (9, 10), may promote allergic lung inflammation *in vivo*.

### DEHP Exposure Enhances the Th2-Stimulating Activity of Splenic cDCs *Ex Vivo*

**Figure 2:**
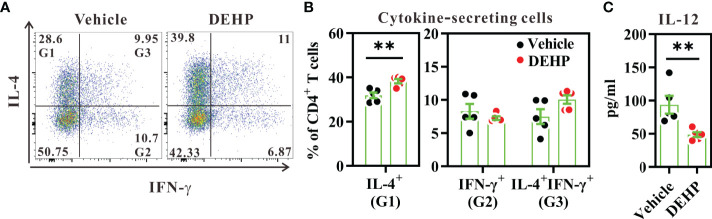
Analysis of cDC function in spleens. BALB/c mice were given oral DEHP, at a dosage of 37 μg/kg BW/day, or 0.33% ethanol in corn oil as vehicle, for 10 d. Purified splenic cDCs from treated mice were co-cultured with naïve CD4^+^ T cells in the presence of anti-CD3 monoclonal antibody for 5 d. **(A)** Representative dot plots show cytokine-secreting cells gated from viable CD4^+^ T cells. **(B)** The percentages of cytokine-secreting cells in individual samples from both groups. **(C)** IL-12 level in CpG1826-stimulated splenic cDCs from treated mice, as analyzed using ELISA. n=5 in each group. Data are representative of two independent experiments. Results are shown as mean ± SEM. **P < 0.01, as assessed using Mann–Whitney U test.

As cDCs govern Th2-mediated lung inflammation (40), we next examined whether chronic exposure to low-dose DEHP skews cDCs to a Th2-stimulating or Th1-suppressing function. Naïve CD4^+^ T cells were co-cultured with purified splenic cDCs from 10-day DEHP-exposed mice or those from the vehicle, in the presence of anti-CD3 stimulation. As shown in **Figures 2A, B**, cDCs from DEHP-exposed mice significantly increased the percentage of IL-4^+^, but not IFN-γ^+^ CD4^+^ T cells. In addition, the purified cDCs from DEHP-exposed mice secreted significantly lower IL-12 levels in response to CpG stimulation, as compared to those from the vehicle group (**Figure 2C**). This suggested the potential impact of DEHP exposure on allergic inflammation, at least in part by modulating cDC differentiation or function.

### DEHP Exposure Alters the Differentiation of Splenic DC Subsets *In Vivo*

**Figure 3:**
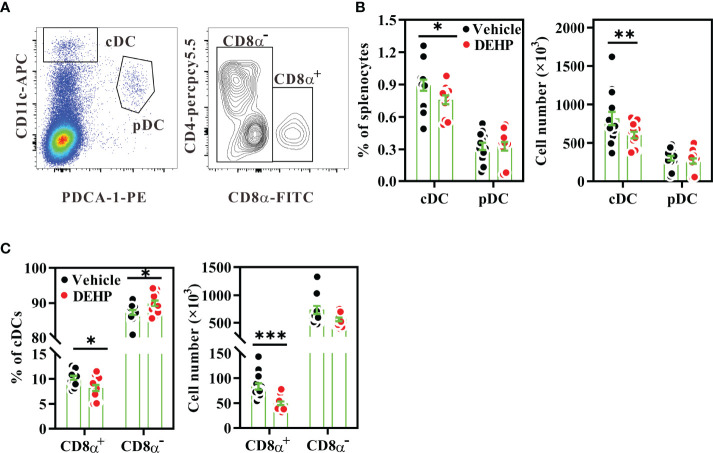
Analysis of frequencies and numbers of splenic DC subsets. BALB/c mice were given oral DEHP, at a dosage of 37 μg/kg BW/day, or 0.33% ethanol in corn oil as vehicle, for 10 d. Splenocytes from the treated mice were assessed for DC subsets using multi-parametric flow cytometry. **(A)** Representative dot plots showing splenic cDCs (CD11c^high^PDCA-1^-^) or pDCs (CD11^low^PDCA-1^+^) (left) and CD8α^+^ (CD4^-^CD8α^+^) and CD8α^-^ (CD4^+^CD8α^-^ and CD4^-^CD8α^-^) DCs gated on cDCs (right) from a vehicle mouse. The percentages or numbers of cDCs, pDCs **(B)**, CD8α^+^ DCs and CD8α^-^ DCs **(C)** in spleens. n=14 mice per group from three independent experiments. Results are shown as mean ± SEM. *P < 0.05, **P < 0.01, and ***P < 0.001 *vs.* vehicle (as assessed using Mann–Whitney U test).

Next, we asked whether altered cDC subsets contributed to the imbalance of Th1 *versus* Th2 activity in the DEHP group. Flow cytometric analysis of splenic DC subsets revealed that DEHP exposure significantly decreased the percentage and number of cDCs (CD11c^high^PDCA-1^-^), but not pDCs (CD11c^low^PDCA-1^+^) in the spleen (**Figures 3A, B**). As CD8α^+^ cDCs preferentially secrete high levels of IL-12 to induce Th1 differentiation, whereas CD8α^-^ cDCs promote Th2 or Th17 differentiation (41), we analyzed the cDC subsets in the spleen. We observed a significant loss in the percentage and absolute number of CD8α^+^ cDCs, but not CD8α^-^ cDCs (a prominent splenic DC subset; **Figure 3C**) in the DEHP group. However, the expression levels of CD86 and MHC class II in both DC subsets were not affected by DEHP exposure (**Supplementary Figure 1**). In addition to CD8α^+^ cDCs, DEHP exposure significantly decreased the absolute number of CD8^+^ T cells, but not CD4^+^ T cells and B cells, in the DEHP group (**Supplementary Figure 2**). These data suggested that DEHP exposure may specifically hamper the differentiation of steady-state CD8α^+^ DCs *in vivo*.

**Figure 4:**
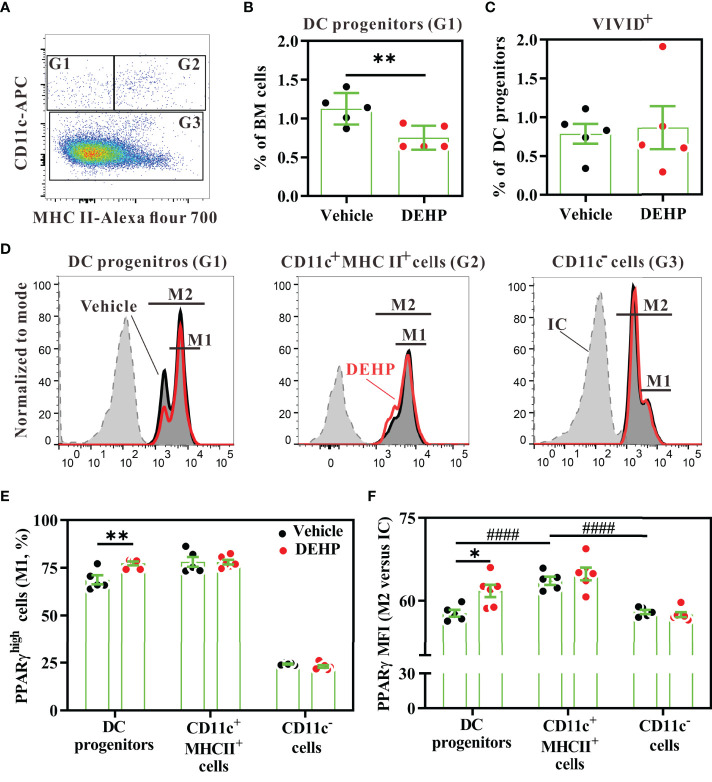
Analysis of PPARγ expression in bone marrow DC progenitors. BALB/c mice were given oral DEHP, at a dosage of 37 μg/kg BW/day, or 0.33% ethanol in corn oil as vehicle, for 10 d. Bone marrow cells from treated mice were analyzed for DC progenitors using multi-parametric flow cytometry. **(A)** Representative dot plots of DC progenitors (G1, CD11c^+^MHC class II^-^), differentiating DCs (G2, CD11c^+^MHC class II^+^), and CD11c^-^ cells (G3) gated from viable Lin^-^ bone marrow cells. **(B)** The percentage of DC progenitors in viable Lin^-^ bone marrow cells. **(C)** The percentage of dead cells (ViViD^+^) in DC progenitors (G1). **(D)** Representative histograms showing intracellular PPARγ expression in each cell subset. **(E)** The frequency of PPARγ^high^ cells (M1) in the gated cells, as shown in **(D)**. **(F)** The fold change of mean fluorescence intensity (MFI) of PPARγ (M2) *versus* isotype control **(IC)** in the gated cells, as shown in **(D)** n = 5 or 6 mice for each group. Results are shown as mean ± SEM. *P < 0.05 and **P < 0.01, as assessed using Mann–Whitney U test. ^####^P < 0.0001, as assessed using one-way ANOVA followed by Dunnett’s multiple comparison test.

As the upstream precursors of splenic DC subsets can be found in the bone marrow (42), we next analyzed the late DC progenitor population (CD45^+^Lin^-^CD11c^+^MHC class II^-^) in the bone marrow, which further differentiates into cDCs and pDCs (43). Flow cytometric analysis revealed that the percentage of late DC progenitors was significantly decreased in the bone marrow of the DEHP group, as compared to that in the vehicle group (**Figures 4A, B**); however, the apoptosis level in the late DC progenitors was similar between these two groups (**Figure 4C**). These data suggested that chronic DEHP exposure may affect splenic cDC differentiation, at least in part by modulating the differentiation of DC progenitors in the bone marrow.

### PPARγ Activity Involves in Altered DC Differentiation in MEHP-Treated FL-DCs

As DEHP is rapidly metabolized to MEHP, a major metabolite in the serum (9, 44), and MEHP is a partial agonist of PPARγ (25), we next investigated whether DEHP exposure altered DC differentiation through the MEHP-PPARγ axis. First, we analyzed PPARγ expression levels in DC progenitors and other cell subsets in the bone marrow. Intracellular flow cytometric analysis revealed that PPARγ protein was expressed in all bone marrow Lin^-^ populations, including late DC progenitors (CD11c^+^MHC class II^-^), differentiating DCs (CD11c^+^MHC class II^+^), and CD11c^-^ subset (CD11c^-^MHC class II^-^ and CD11c^-^MHC class II^+^) (**Figure 4D**). The expression level of PPARγ was significantly higher in the differentiating DCs than in late DC progenitors or the CD11c^-^ subset (**Figure 4F**). However, DEHP exposure significantly increased the PPARγ^high^ cell percentage and expression level in the late DC progenitors, but not in the other two subpopulations (**Figures 4E, F**). These data suggested that chronic and low-dose DEHP exposure may specifically increase PPARγ activity in late DC progenitors in the bone marrow.

**Figure 5:**
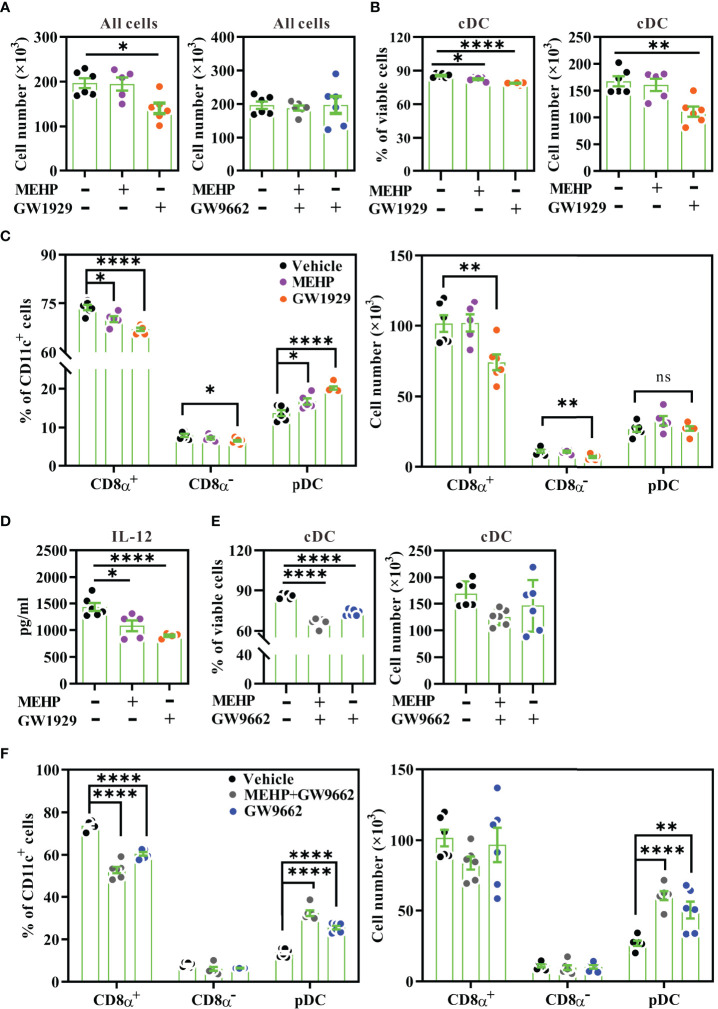
DC subset analysis in Flt3L-induced bone-marrow culture. Bone marrow cells from BALB/c mice were treated with various concentrations of MEHP (20 μM), GW1929 (4 μM), and/or GW9662 (4 μM) for 7 d, in the presence of rmFlt3L. Day-8 FL-DCs were harvested for subset analysis using multi-parametric flow cytometry. The gating strategy is shown in **Supplementary Figure 3**. **(A)** Total cell numbers of day-8 FL-DCs treated with MEHP, GW1929, and/or GW9662. **(B)** The percentages and cell numbers of cDCs gated from viable FL-DCs treated with MEHP or GW1929. **(C)** The percentages and cell numbers of CD8α^+^ DCs, CD8α^-^ DCs, and pDCs gated from CD11c^+^ cells treated with MEHP or GW1929. **(D)** IL-12 levels of treated FL-DCs after stimulation with CpG1826 for 24 h. **(E)** The percentages and cell numbers of cDCs gated from viable FL-DCs treated with MEHP and/or GW9662. **(F)** The percentages and cell numbers of CD8α^+^ DCs, CD8α^-^ DCs, and pDCs gated from CD11c^+^ cells treated with MEHP and/or GW9662. n=5 or 6 for each treatment. Results are represented as mean ± SEM. *P < 0.05, **P < 0.01, and ****P < 0.0001, as assessed using one-way ANOVA followed by Dunnett’s multiple comparison test. ns, non-significant.

Next, we investigated whether the MEHP-PPARγ activation axis altered DC subset differentiation using Flt3L-induced bone-marrow culture, as Flt3L-differentiated dendritic cells (FL-DCs) comprise of CD8α^+^ DCs, CD8α^-^ DCs, and pDCs, which are similar to those normally found in mouse spleens (37). To mimic chronic exposure to DEHP in humans, bone marrow cells were treated with MEHP for 7 days, starting from day 1, and the DC subsets were analyzed on day 8. GW1929, a potent selective PPARγ agonist, was used as a positive control for PPARγ activation. During the differentiation period, daily treatment with GW1929, at a concentration of 4 μM, significantly decreased the absolute number of FL-DCs harvested from day-8 culture (**Figures 5A**, **left**). The significant loss of DC subset upon GW1929 treatment was that of cDCs (CD11c^+^CD45RA^-^), including CD8α^+^ DCs (CD11b^low^CD24^high^) and CD8α^-^ DCs (CD11b^high^CD24^low/-^), but not pDCs (CD11c^+^CD45RA^+^) (**Supplementary Figure 3A**; **Figures 5B, C**). Similar to GW1929, MEHP had a moderate effect on CD8α^+^ DC differentiation. MEHP decreased the percentages of CD8α^+^ DCs and cDCs, but not CD8α^-^ DCs. Due to the decreased percentage of the cDC subset, there was a considerable change in the relative proportion of pDCs in MEHP-treated conditions; however, there was no significant difference in the absolute number of pDCs between vehicle and MEHP treatment (**Figures 5B, C**). In addition, both GW1929 and MEHP treatment significantly decreased IL-12 production in CpG1826-stimulated FL-DCs (**Figure 5D**). On the other hand, MEHP treatment did not affect CD86 and MHC class II expression in CD8α^+^ DCs (**Supplementary Figure 3B**). These data suggested that the effect of MEHP on the disturbance of cDC differentiation is associated with PPARγ involvement.

**Figure 6:**
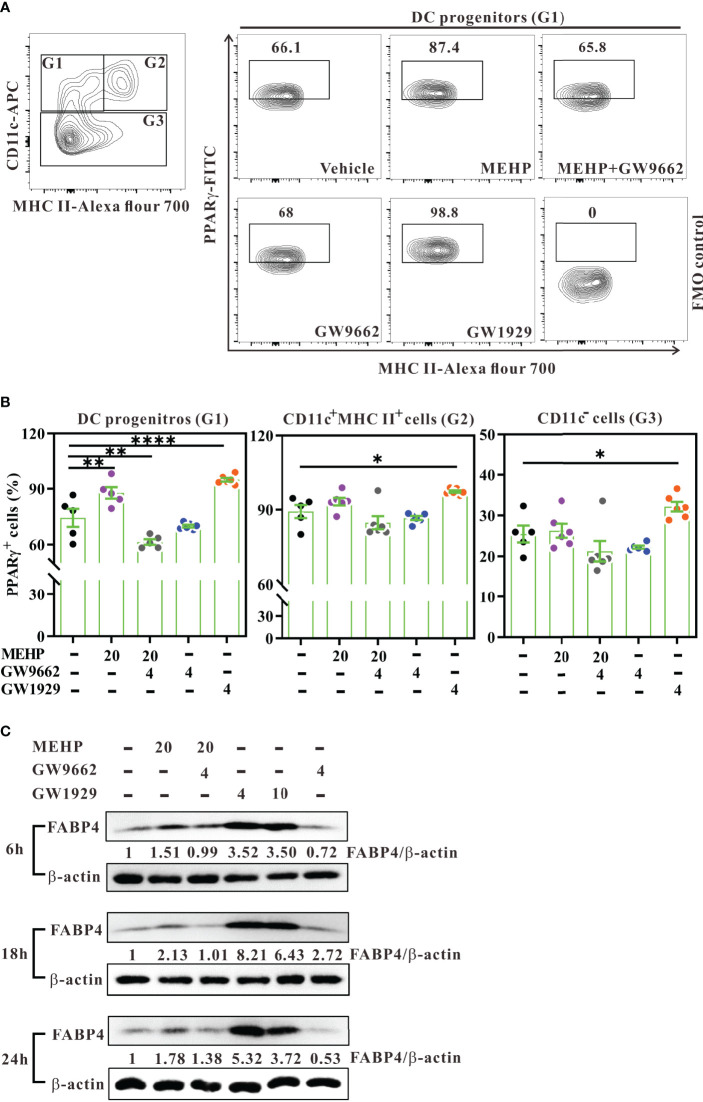
Expression and activity of PPARγ in Flt3L-induced bone-marrow culture. **(A)** Day-3 Flt3L-differentiated bone marrow cells were treated with various concentrations (μM) of MEHP, GW1929, and/or GW9662 for 6 h and then analyzed for PPARγ expression using multi-parametric flow cytometry. Representative contour plots showing intracellular PPARγ expression in DC progenitors (G1, CD11c^+^MHC class II^-^) from treated cells gated on viable Lin^-^ bone marrow cells. PPARγ^+^ cells were gated against fluorescence minus one (FMO) control. **(B)** Percentages of PPARγ^+^ cells in each subset. n=5 or 6 mice for each group. Results are represented as mean ± SEM. *P < 0.05, **P < 0.01, and ****P < 0.0001, as assessed using one-way ANOVA followed by Dunnett’s multiple comparison test. **(C)** Estimation of protein levels of FABP4 and β-actin in day-3 Flt3L-differentiated bone marrow cells treated with various conditions for 6, 18, and 24 h, using western blot. Data are representative of two experiments. Results are shown as fold enrichment by normalizing the relative ratio of FABP4 *versus* β-actin to vehicle control.

We next examined whether GW9662, a selective PPARγ antagonist, reversed the effect of MEHP on DC differentiation. Daily treatment with GW9662 did not alter the absolute numbers of day-8 FL-DCs (**Figure 5A****, right**) or the cDC subset (**Figure 5E****; right**). However, we unexpectedly observed that GW9662 treatment alone significantly increased the percentage and cell number of pDCs (**Figure 5F**). The decreased percentages of cDCs or CD8α^+^ DCs upon GW9662 treatment were due to the relative increase in the proportion of pDCs (**Figures 5E, F****; left panels**). Co-treatment of MEHP with GW9662 had a similar effect as treatment with GW9662 alone. This finding suggested that inhibition of endogenous PPARγ activity may alter DC subset differentiation.

### MEHP Alters cDC Differentiation, at Least in Part in a PPARγ Activation-Dependent Manner

To ensure that PPARγ activity was activated by MEHP, day-3 Flt3L-differentiating bone marrow cells were treated with MEHP, GW1929, and/or GW9662, and then assessed for the expression of PPARγ or FABP4, a PPARγ-regulated downstream gene (45). Initially, western blot analysis showed that MEHP treatment for 6 or 18 hours only showed moderate enhancement of PPARγ expression, whereas GW1929 did increase PPARγ expression upon treatment at the concentration of 4 μM (**Supplementary Figure 4**). Next, we analyzed intracellular PPARγ expression in DC progenitors using multi-parametric flow cytometry. After 6 hours of treatment, both MEHP and GW1929 significantly increased the percentage of PPARγ^+^ cells in DC progenitors, and this effect was fully inhibited by the antagonist GW9662 (**Figures 6A, B****; left panel**). Interestingly, MEHP did not affect PPARγ expression in differentiating DCs (CD11c^+^MHC class II^+^) or CD11c^-^ subsets, in Lin^-^ bone marrow cells (**Supplementary Figures 5A, B****;** **Figure 6B****, middle and right panels**). However, GW1929 significantly increased PPARγ expression in these three subsets (**Figure 6B**), possibly because its potency is approximately equivalent to that of rosiglitazone and much higher than that of MEHP (25, 46). In addition, western blot analysis revealed that MEHP upregulated FABP4 expression (approximately 1.5- to 2.1-fold) at 6, 18, and 24-hour post-treatment, whereas this MHEP effect was reversed by GW9662 (**Figure 6C**). In contrast, GW1929 dramatically upregulated FABP4 expression from 6 to 24 h post-treatment in Flt3L-differentiating bone marrow cells (**Figure 6C**).

To further ensure that MEHP displayed similar effect on inflammatory DC differentiation *via* PPARγ activation, GM-CSF bone marrow culture were treated with MEHP, GW1929, and/or GW9662 for 7 days. As Helft J et al. clearly revealed that GM-CSF-culture produced MHC class II^high^ dendritic cells (GM-DCs) and MHC class II^int^ macrophages (GM-Macs) (47), we separately analyzed the effect of MEHP on DC and Mac subsets using multi-parametric flow cytometry. As shown in **Supplementary Figure 6**, MEHP treatment significantly decreased the percentage and number of CD115^-^ GM-DCs, but not the CD115^+^ GM-Macs subset (**Supplementary Figures 6C, D**). Interestingly, MEHP significantly increased the percentage and number of the CD11b^int^MHC class II^int^ subset in a dose-dependent manner (**Supplementary Figure 6E**). The effect of MEHP on CD115^-^ GM-DCs and the CD11b^int^MHC class II^int^ subset is similar to that of GW1929 (PPARγ agonist), but reverse to that of GW9662 (PPARγ antagonist) (**Supplementary Figures 6C, E**). Taken together, analysis of GM-CSF-induced cell culture revealed that MEHP treatment affects DC differentiation, but not Mac differentiation, thus supporting the findings observed in the FL-DCs. These results suggested that chronic DEHP exposure may alter cDC differentiation, at least in part through the MEHP-PPARγ activation axis.

## Discussion

**Figure 7:**
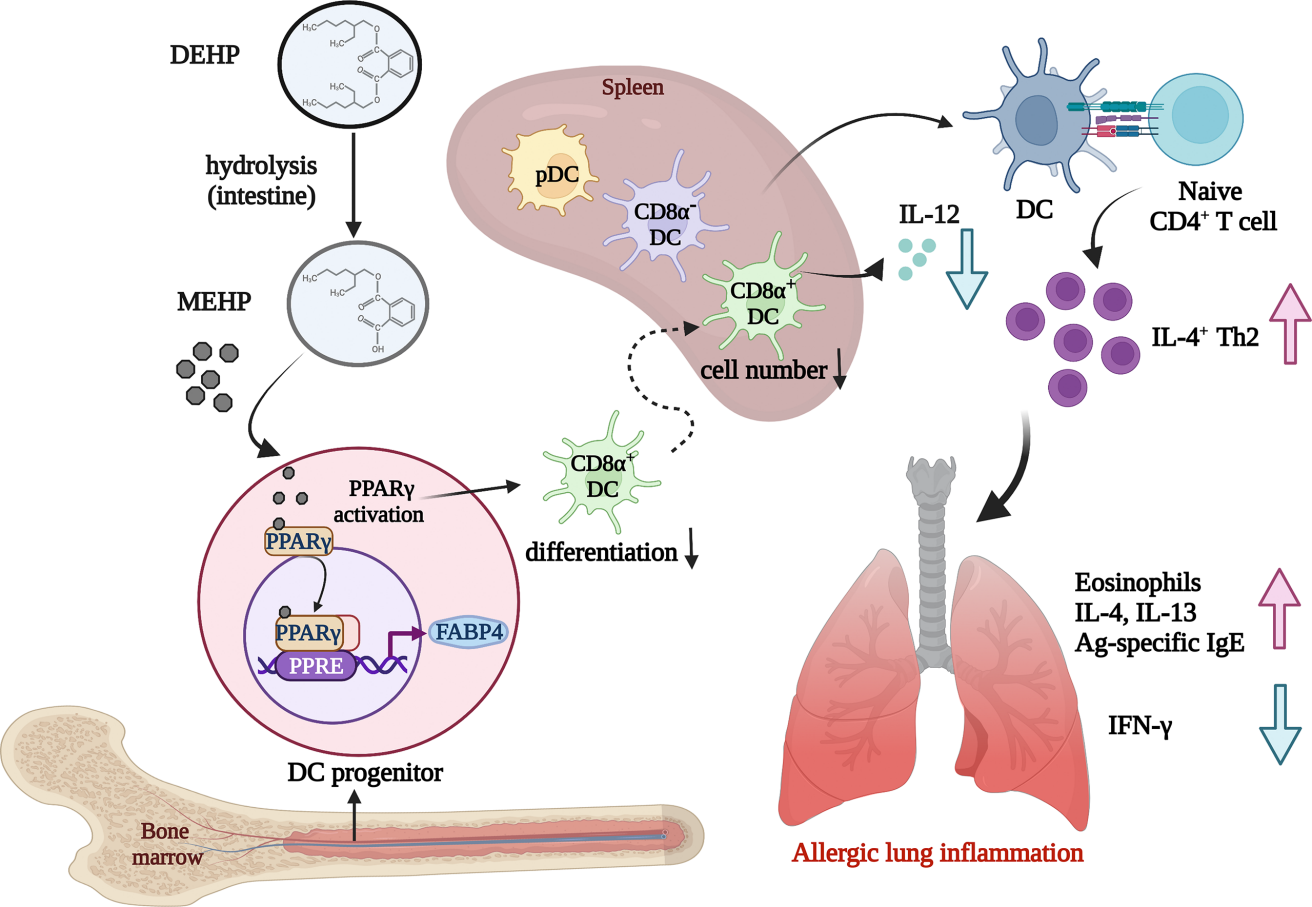
Schematic representation showing the effect of environmental DEHP exposure on allergic lung inflammation. Chronic and low-level exposure to DEHP, a common plasticizer and environmental endocrine disruptor, may result in altered homeostasis of DC subsets, particularly block CD8α^+^ DC differentiation, through the MEHP-PPARγ activation axis. The loss of CD8α^+^ DCs, a main IL-12 cellular source, may contribute to the enhanced allergic lung inflammation, at least in part through Th2 polarization in DEHP-exposed mice. Image created with BioRender.com.

The present study provides evidence that mimicking human exposure to DEHP at physiologically relevant doses alters cDC differentiation, leading to an enhanced allergic response. To the best of our knowledge, this is the first study to demonstrate that MEHP hinders CD8α^+^ DC differentiation, at least in part through PPARγ activation, in mice and in a Flt3L-supplemented culture system (see **Figure 7** for schematic presentation). In addition, the *in vitro* pharmacological experiments shown in this study suggest that PPARγ inactivation may alter pDC homeostasis. This study provides a potential mechanistic link between ubiquitous phthalate exposure and PPAR-regulated DC differentiation in allergic lung inflammation.

Our purpose was to study the immunomodulatory effect (low-level but continuous exposure), rather than toxic effect (one single high-dose exposure) of DEHP on allergic asthma. Therefore, the *in vivo* dose of DEHP selected in this study was lower than the current human TDI (36, 39) and can be considered an environmentally relevant dose. The *in vitro* concentration of MEHP used in this study was up to 20 μM, which is between the highest MEHP level (26.47 μg/ml, ~95 μM) and median level (0.58 μg/ml, ~2 μM) that had been reported in plasma with endometriosis (48). The *in vitro* concentration of MEHP used in this study can be considered as low-level exposure. In addition, in contrast to long-term treatment, 24-hour short-term treatment with MEHP did not show any effect on the differentiation of FL-DCs *in vitro* (data not shown). Taken together, we expected that chronic exposure to DEHP at environmentally relevant doses would not be toxic, but would rather modulate cDC differentiation, which in turn would modify the magnitude of the resultant allergic response.

Cell type-specific knockout strategies demonstrate that DCs, macrophages, and CD4^+^ T cells express PPARγ and coordinately drive pathogenic type-2 lung inflammation (31, 49). Therefore, DEHP exposure is expected to modulate the function or homeostasis of these immune cell subsets *via* MEHP. Our results suggest that MEHP is a bioactive metabolite of DEHP *in vivo*, because MEHP at the same TDI dose exerts similar effects on OVA-induced lung inflammation, in terms of infiltrated cell types and their respective cytokine generation. The *in vitro* results showed that long-term treatment of Flt3L-induced bone marrow culture with MEHP significantly decreased the number of CD8α^+^ DCs, supporting the observation of altered splenic DC homeostasis in DEHP-treated mice. In addition, the proportion of PPARγ^high/+^ DC progenitors significantly increased in the bone marrow of DEHP-treated mice or in MEHP-conditioned Flt3L-induced bone marrow culture. Taken together, the present study suggests that MEHP is the main bioactive metabolite of DEHP that modulates allergic inflammation *in vivo*.

PPARγ is a ligand-activated nuclear receptor that regulates fatty acid storage, glucose metabolism, and immunity (50, 51). In addition to promoting the polarization of type 2 macrophages (52) and enhancing the accumulation of adipose tissue regulatory T-cells at inflammatory sites (53), pharmacological PPARγ activation also modulates DCs to display immune suppressive functions, as demonstrated by GM-CSF bone marrow culture in mice (54), human monocyte-derived DC differentiation (26, 55), and *in vivo* models (34, 51). In the present study, the basal expression of PPARγ and FABP4 supports the endogenous activity of PPARγ in CD45^+^Lin^-^ bone marrow cells. Unexpectedly, we observed that *in vitro* MEHP treatment or *in vivo* DEHP exposure increased PPARγ expression only in DC progenitors (Lin^-^CD11c^+^MHC class II^-^), but not in other Lin^-^ subpopulations (CD11c^+^MHC class II^+^ and CD11c^-^ subsets), although all Lin^-^ cells expressed PPARγ. However, *in vitro* GW1929 (agonist) treatment enhanced PPARγ expression in all three subsets (Lin^-^) in the bone marrow. A possible reason may be that MEHP is a partial PPARγ agonist whose efficacy is much lower than that of rosiglitazone (25) or GW1929 (56), the full agonists of PPARγ. Consistent with the effect on PPARγ expression, GW1929 also had a more dramatic inhibitory effect on CD8α^+^ DC differentiation than MEHP, in Flt3L-induced bone marrow culture. These results suggest that chronic and low-level exposure to DEHP may block CD8α^+^ DC differentiation, at least in part, through PPARγ activation in a steady state. The specific role(s) that PPARγ plays in DC progenitors remains unclear, and the underlying mechanism needs to be investigated in depth.

The homeostasis of CD8α^+^ DCs seems to be sensitive to modulation by chronic DEHP exposure at the human TDI level. Our recent study demonstrated that chronic and low-dose maternal exposure to DEHP enhances allergic lung inflammation in young offspring, through at least four generations (21). This ancestral DEHP exposure leads to decreased numbers of splenic CD8α^+^ DCs and bone marrow DC progenitors, at least in part through epigenetic modification in DCs (21). Maternal DEHP-mediated DC homeostasis alteration has been shown to be related to apoptosis in DC progenitors and splenic DCs from offspring (21), whereas the present study shows that DEHP exposure limits the differentiation of CD8α^+^ DCs from the bone marrow, instead of apoptosis. In the context of OVA sensitization in the present study, we speculated that Ag-specific Th2 cells activated by splenic DCs could migrate to allergen-challenged lung (57), leading to enhanced allergic lung inflammation in DEHP-treated mice. Due to the complexity of DC network and biology, the detailed mechanisms regarding the interaction between environmental endocrine disruptors and immunity await further in-depth studies.

There are two limitations in this study. The first one is that the effect of DEHP/MEHP was not analyzed in inhaled-allergen asthma model as airway DC subsets, comprising of resident, migratory, and inflammatory DCs, also play important roles in establishment of airway allergic inflammation (58, 59). The other is that adoptive transfer experiment of MEHP-conditioned FL-DCs was not performed. In summary, the current study provides immunological evidence supporting a causal relationship between environmental DEHP exposure and allergy development. Our findings also provide a potential link between plasticizers, PPARγ, and cDC homeostasis. This study also provides a rational basis for elucidating the effect of phthalate exposure on tumor immunity and vaccine response, in which CD8α^+^ DCs play important roles.

## Data Availability Statement

The raw data supporting the conclusions of this article will be made available by the authors, without undue reservation.

## Ethics Statement

The animal study was reviewed and approved by The Institutional Animal Care and Use Committee of Kaohsiung Medical University (permit numbers: 104115, 109075).

## Author Contributions

J-LS and S-KH conceived and designed the experiments; H-HT, C-YL, S-TW, H-HS, T-HW, H-EW, and Y-WC performed the experiments and analyzed the data; H-HT and J-LS wrote the manuscript; J-LS, S-KH, and EMT revised the manuscript. All authors contributed to the article and approved the submitted version.

## Funding

This work was supported by funds KMU-N102171, KMU-TP104A05, and KMU-N105181 from Kaohsiung Medical University; MOST 108-2314-B-037-001 and MOST 110-2320-B-037-024 from the Ministry of Science and Technology, Taiwan; EM-109-PP-10 from National Health Research Institutes; KMU-TC108A01-1 from the Research Center for Environmental Medicine of Kaohsiung Medical University, Ministry of Health and Welfare (Project No. 11068), and the Research Center for Environmental Medicine of Kaohsiung Medical University from the Featured Areas Research Center Program, within the framework of the Higher Education Sprout Project by the Ministry of Education (MOE) of Taiwan.

## Conflict of Interest

The authors declare that the research was conducted in the absence of any commercial or financial relationships that could be construed as a potential conflict of interest.

## Publisher’s Note

All claims expressed in this article are solely those of the authors and do not necessarily represent those of their affiliated organizations, or those of the publisher, the editors and the reviewers. Any product that may be evaluated in this article, or claim that may be made by its manufacturer, is not guaranteed or endorsed by the publisher.

## Abbreviations

BW, body weight; BMDC, bone marrow-derived dendritic cell; BALF, bronchoalveolar lavage fluid; DEHP, di-(2-ethylhexyl) phthalate; MEHP, mono-(2-ethylhexyl) phthalate; FABP4, fatty acid-binding protein 4; Flt3L, Fms-like tyrosine kinase 3 ligand; FL-DC, Flt3L-differentiated dendritic cell; GM-CSF, granulocyte macrophage colony-stimulating factor; GM-DC, GM-CSF-differentiated dendritic cell; OVA, ovalbumin; PPARγ, peroxisome proliferator-activated receptor gamma; pDCs, plasmacytoid dendritic cells; TDI, tolerable daily intake.
