# Supplementary material for: Di-(2-ethylhexyl) Phthalate Promotes Allergic Lung Inflammation by Modulating CD8α+ Dendritic Cell Differentiation via Metabolite MEHP-PPARγ Axis
Source: Front Immunol. 2022 May 19;13:581854. doi: 10.3389/fimmu.2022.581854 (PMC9160748; doi:10.3389/fimmu.2022.581854)
Supplement: Supplementary file 1 [file DataSheet_1.pdf]

Supplementary Material

GM-CSF-differentiated Dendritic Cell (GM-DC) Treatment

Bone-marrow cells were treated with MEHP, GW9662 (PPARγ antagonist; Tocris, Bristol, UK), or GW1929 (PPARγ agonist; Sigma-Aldrich) at various concentrations or with 0.1% ethanol (vehicle control) at the beginning of day 1 of culture in medium containing recombinant murine GM-CSF (10 ng/ml, PeproTech, Rocky Hill, NJ) and 2-mercaptoethanol (50 μM, Sigma-Aldrich). The medium containing rmGM-CSF and/or chemicals or 0.1% ethanol was refreshed on days 4 and 6. On day 8, the cells were harvested for phenotype analysis using flow cytometry (LSRII; BD Biosciences). The fluorochrome-labeled antibodies included eF450-conjugated anti-CD11c (N418; eBioscience), PerCP-cy5.5-conjugated anti-CD11b (M1/70; BioLegend), Alexa Fluor 488-conjugated anti-CD115 (AFS98; BioLegend), Alexa Fluor 700-conjugated anti-MHC II (M5/114.15.2; eBioscience), and Live/Dead™ fixable Red (Invitrogen).

Supplementary Figures

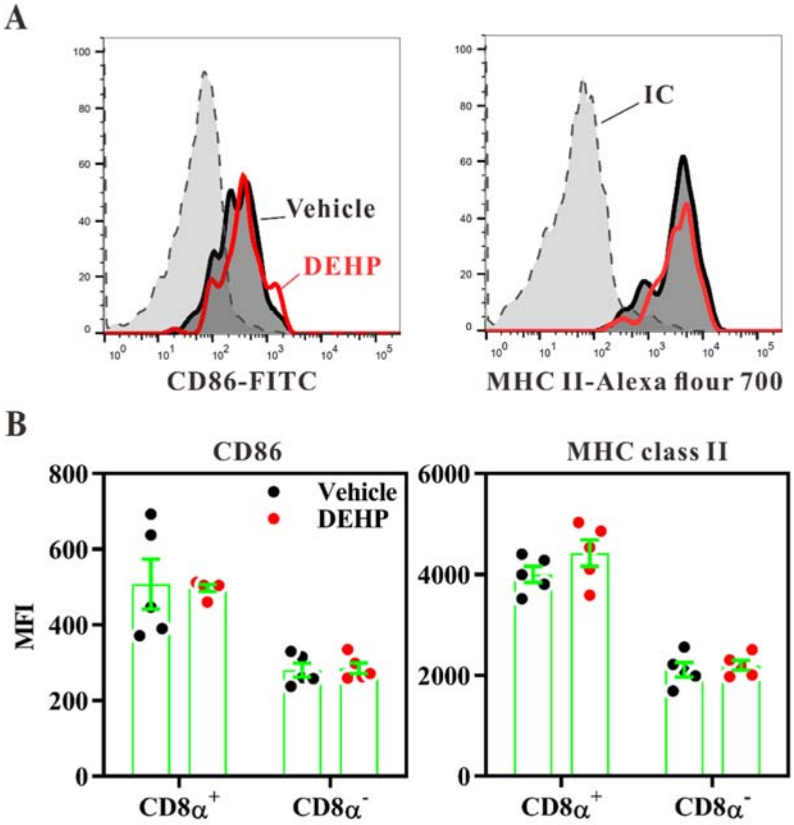

Supplementary Figure 1. Phenotypic analysis of splenic DC subsets. BALB/c mice were given oral DEHP, at a dosage of 37 µg/kg BW/day, or 0.33% ethanol in corn oil as vehicle, for 10 d. Splenocytes from treated mice were analyzed for DC markers using flow cytometry. **(A)** Representative histograms showing the expression levels of CD86 and MHC class II in splenic cDC subsets. **(B)** The mean fluorescence intensity (MFI) of CD86 or MHC class II in splenic cDC subsets. n=5 mice in each group. Results are represented as mean ± SEM.

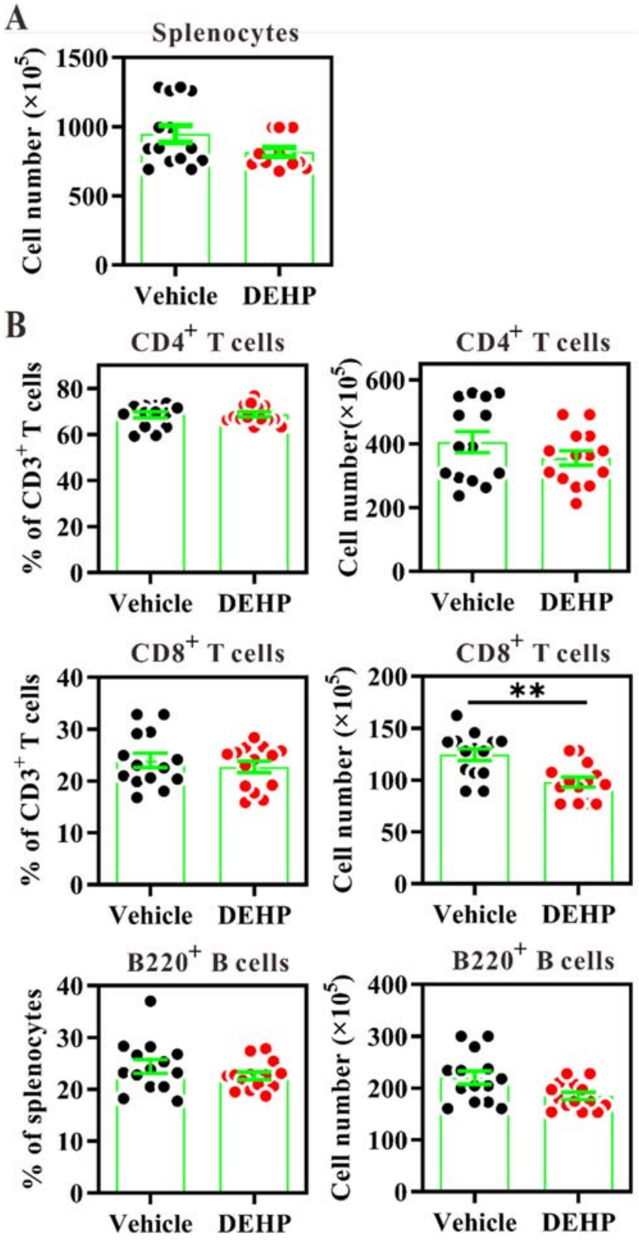

Supplementary Figure 2. Analysis of frequencies and numbers of splenic lymphocyte subsets. BALB/c mice were given oral DEHP, at a dosage of 37 μg/kg BW/day, or 0.33% ethanol in corn oil as vehicle, for 10 d. Splenocytes from the treated mice were assessed for lymphocyte subsets using multi-parametric flow cytometry. **(A)** Total cell numbers of splenocytes from treated mice. **(B)** The frequencies and cell numbers of $CD4^{+}$ T cells ($CD3^{+}CD4^{+}CD8^{-}B220^{-}$), $CD8^{+}$ T cells ($CD3^{+}CD4^{-}CD8^{+}B220^{-}$), and B cells ($CD3^{-}B220^{+}$) in spleens. n=14 mice in each group. Results are represented as mean ± SEM from three independent experiments. **P<0.01, as assessed using Mann–Whitney U test.

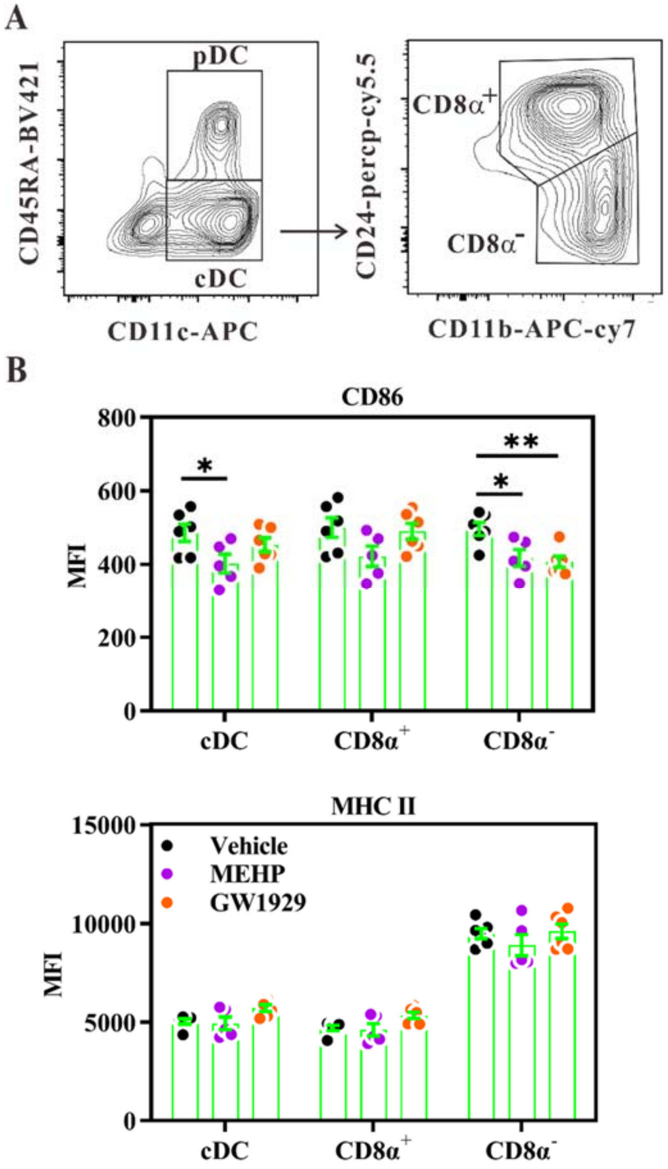

Supplementary Figure 3. Phenotypic analysis of DC subsets in Flt3L-induced bone-marrow culture. Bone marrow cells from BALB/c mice were treated with various concentrations of MEHP (20 μM) or GW1929 (4 μM) for 7 d, in the presence of rmFlt3L. Day-8 FL-DCs were harvested for phenotypic analysis of DC subsets using multi-parametric flow cytometry. **(A)** Representative contour plots showing the gating strategy from left to right. **(B)** The mean fluorescence intensity (MFI) of CD86 and MHC class II in cDCs ($CD11c^{+}CD45RA^{-}$) gated from viable cells, as well as, $CD8\alpha^{+}$ cDCs ($CD11b^{+}CD24^{+}$) and $CD8\alpha^{-}$ cDCs ($CD11b^{+}CD24^{-}$) gated from cDCs. n=5 or 6 in each treatment. Results are represented as mean ± SEM. *P<0.05 and **P<0.01, as assessed using one-way ANOVA followed by Dunnett's multiple comparison test.

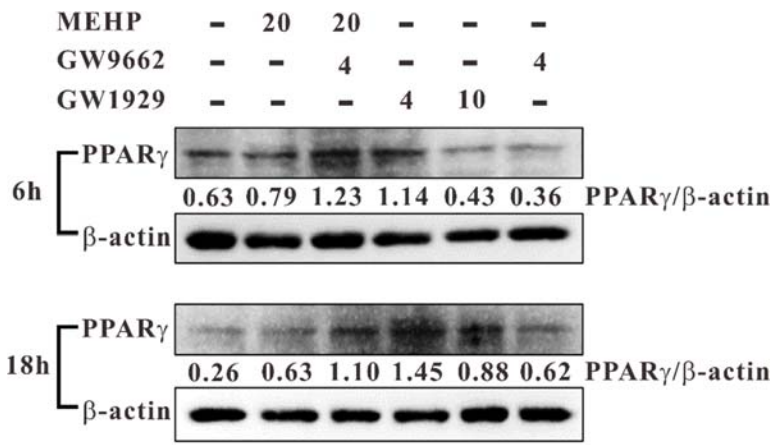

Supplementary Figure 4. PPARγ expression in Flt3L-induced bone-marrow culture. Estimation of protein level of PPARγ in day-3 Flt3L-differentiated bone marrow cells treated with various conditions for 6 and 18 h, using western blot. Data are representative of two experiments. Results are shown as fold enrichment by normalizing the relative ratio of PPARγ *versus* β-actin to vehicle control.

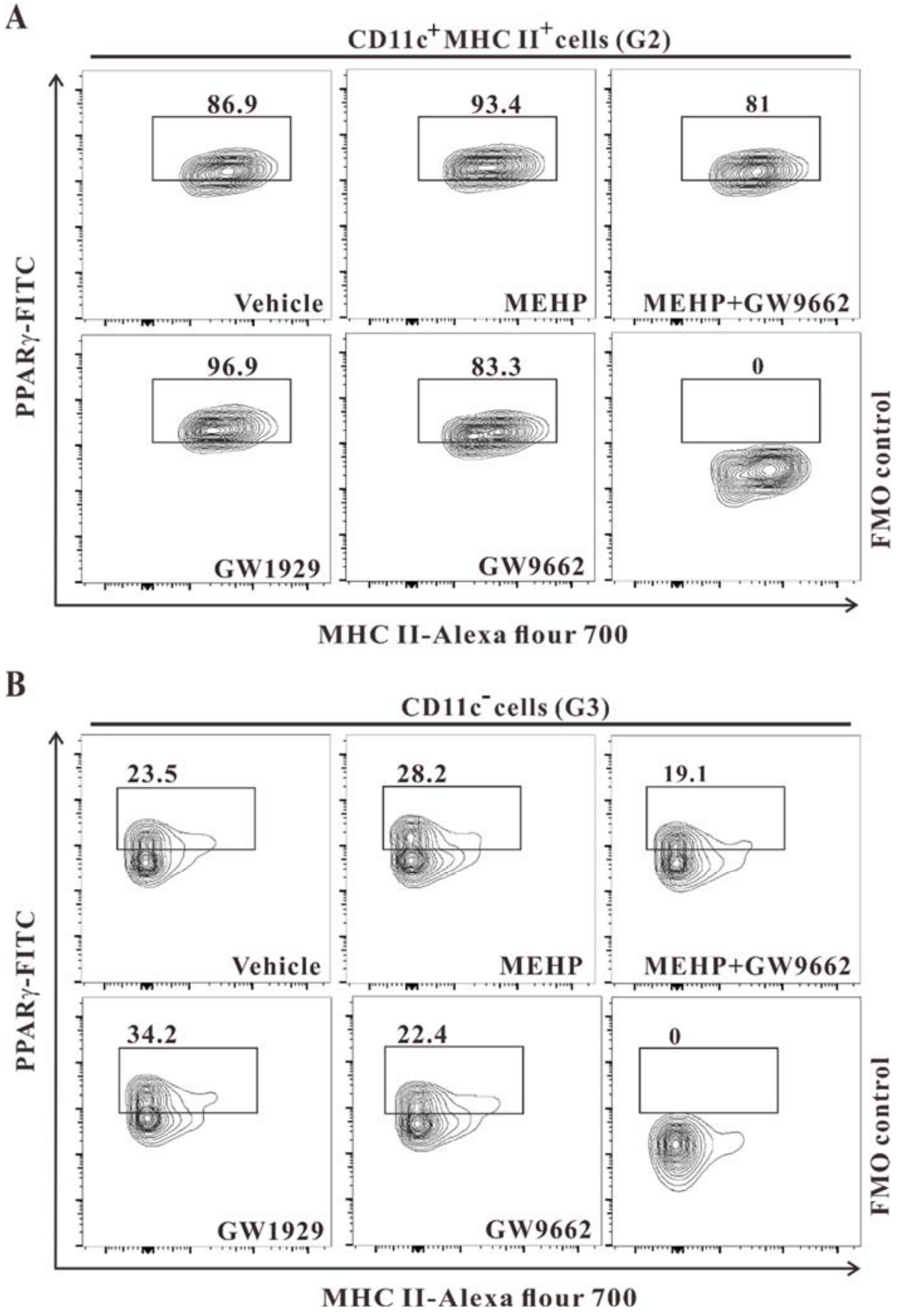

Supplementary Figure 5. Flow cytometric analysis of PPARγ expression in Flt3L-induced bone-marrow culture. Day-3 Flt3L-differentiated bone marrow cells were treated with various conditions for 6 h, as shown in **Fig. 6B**. Representative contour plots showing intracellular PPARγ expression in differentiating DCs (G2 as shown in **Fig. 6A**, CD11c^{+}MHC class II^{+}) or CD11c^{-} cells (G3 as shown in **Fig. 6A**) from treated cells gated on viable Lin^{-} bone marrow cells. PPARγ^{+} cells were gated against fluorescence minus one (FMO) control.

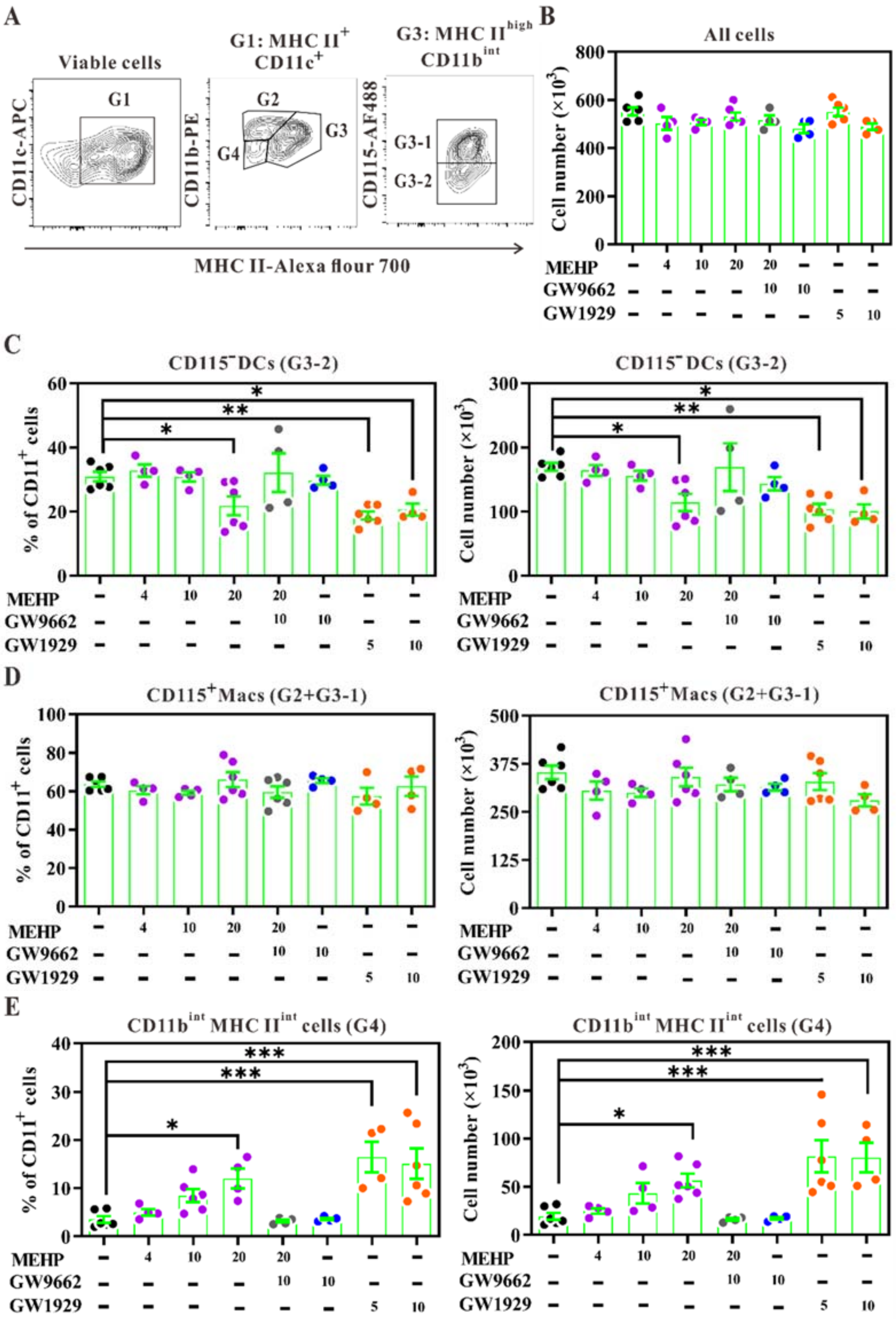

Supplementary Figure 6. Phenotypic analysis of dendritic cell (DC) and macrophage (Mac) subsets in GM-CSF-induced bone-marrow culture. Bone marrow cells from BALB/c mice were treated with various concentrations (μM) of MEHP or GW1929 (as indicated) for 7 d, in the presence of rmGM-CSF. Day-8 GM-DCs were harvested for phenotypic analysis of cell subsets using multi-parametric flow cytometry. **(A)** Representative contour plots showing the gating strategy from left to right. **(B)** Total cell numbers of day-8 GM-CSF-induced bone-marrow culture. **(C)** The frequency and cell

number of CD115$^-$ DCs (gate G3-2). The CD11c$^+$MHC class II^{high}CD11b$^+$CD115$^-$ cells were defined as CD115$^-$ DCs. **(D)** The frequency and cell number of CD115$^+$ Macs (gate G2 and gate G3-1). The Macs included two subsets: CD11c$^+$MHC class II^{low}CD11b^{high} (gate G2) and CD11c$^+$MHC class II^{high}CD11b$^+$CD115$^+$ (gate G3-1) cells. **(E)** Frequency and cell number of the CD11b^{int}MHC class II^{int} subset (gate G4); n=4–6. Results are shown as mean ± SEM from two independent experiments. **P<0.01, as assessed using Mann–Whitney U test. *P<0.05; **P<0.01; ***P<0.001, as assessed using one-way ANOVA followed by Dunnett's multiple comparison test.
